# Germany: a fair balance between scientific freedom and data subjects’ rights?

**DOI:** 10.1007/s00439-018-1912-1

**Published:** 2018-08-16

**Authors:** Fruzsina Molnár-Gábor

**Affiliations:** Heidelberg Academy of Sciences and Humanities, Heidelberg, Germany

## Abstract

With the German Bundestag’s adoption of the Data Protection Adaptation and Implementation Act EU (DSAnpUG-EU) on 30 June 2017, the adaptation of German law to the General Data Protection Regulation (GDPR) has begun (Gesetz zur Anpassung des Datenschutzrechts an die Verordnung (EU) 2016/679 und zur Umsetzung der Richtlinie (EU) 2016/680 (Datenschutz-Anpassungs- und -Umsetzungsgesetz—DSAnpUG-EU) v. 30. Juni 2017, BGBl. 2017 I p. 2097 et seq.). Despite being directly binding on all EU member states, the GDPR does not render national data protection provision obsolete—they are covered by the GDPR’s opening clauses which include regulatory mandates and room for derogation. This creates considerable need for national legislative adaptation. Art. 1 DSAnpUG-EU contains the necessary amendments to the Federal Data Protection Law (BDSG(neu)), thus creating the second major building block of future German data protection alongside the GDPR itself. Nevertheless, there are still numerous sector-specific regulations in other federal laws and the data protection laws of the 16 states also need amendments. Adjustment in Germany is well on its way, but implementation in general is still ongoing, with further consequences for data processing and sharing.

## Introduction

When considering genomic data sharing for scientific research purposes in the form of transfers to third countries, we must first clarify the scope of applicability of the data protection regulations.

The GDPR defines “genetic data” as personal data relating to the inherited or acquired genetic characteristics of a natural person which result from the analysis of a biological sample from the natural person in question, in particular chromosomal, DNA, or RNA analysis, or from the analysis of another element enabling equivalent information to be obtained (recital 34 GDPR).

Neither the BDSG(neu) nor the GDPR defines the term “transfer”. While the broad definition of processing in Art. 4 No. 2 GDPR includes “disclosure by transmission”, transfers are actually regulated by Chapter V. This muddies the waters as to whether the two terms are equivalent. It is at least clear that processors under the GDPR are not third parties (Art. 4 No. 10 GDPR), irrespective of whether they are in the EU, but are instead recipients within the meaning of Art. 4 No. 9 GDPR, to whom data are “disclosed”. Since Chapter V only refers to “transfer” as such, it also applies to the disclosure of personal data to a processor in a third country. Thus, “transfer” must be understood as any disclosure of personal data to a recipient in a third country, whereby neither the type of disclosure nor the recipient’s third-party status is decisive.^1^

The BDSG(neu) also contains no definition of “scientific research purposes”. According to the GDPR, “research” must be interpreted broadly, including basic, applied, and privately financed research, as well as studies conducted in the public interest in the field of public health (recital 113 in connection with recital 159 GDPR).^2^ In the field of clinical studies, the relationship of scientific research to the subject matter of the German Medicines Act^3^ (in particular Art. 40 (2) (a) AMG) and to the EU Regulation No. 536/2014 on clinical trials of medicinal products for human use^4^ has not yet been finally clarified. However, since the Regulation No. 536/2014 refers in Art. 93 to the Data Protection Directive (DPD, the GDPR’s predecessor in matters of personal data processing), and the GDPR mentions clinical studies alongside scientific research (recital 161 GDPR), it can be assumed that the privileged treatment of scientific research also includes clinical studies.^5^ Nevertheless, it remains to be seen whether the national legislator will produce more specific regulations by adapting the AMG’s sector-specific data protection regulations.

There is a two-stage permissibility test to determine whether genomic data can be transferred to recipients in the third countries outside of the EU for scientific research purposes.^6^ This system corresponds to that of Art. 4 b (2) et seq. of the previous Federal Data Protection Law: the first stage asks whether the transfer as-such is permitted irrespective of the reference to a third country—i.e., whether the planned transfer complies in principle with the general substantive requirements of the GDPR in connection—where relevant—with the BDSG(neu). In the case of genetic data transfers, Art. 9 (1) GDPR initially provides for a so-called prohibition in principle of any processing and thus any transfer, while allowing certain exceptions. To transfer genomic data, one of the permissions for processing provided by the GDPR—potentially in connection with BDSG(neu)—must thus be invoked. The permissibility of said processing for the specific purpose of scientific research must also be considered here, as data controllers and processors must ensure that, regardless of their specific purpose, they provide appropriate safeguards to ensure that the rights and freedoms of data subjects are not unduly restricted.

Once this first stage permissibility test has been passed, the controller responsible for data processing can examine in the second stage whether conditions for the transfer of genomic data to third countries have been fulfilled. In principle, this can only occur where the requirements of Chapter V of the GDPR are fulfilled. This chapter creates a final catalog of permissions for the transfer of personal data to a recipient in a third country or to international organizations.^7^ These requirements extend the protection of personal data throughout the EU, ensuring that the provisions of Chapter V are always applied, such that the level of protection cannot fall below the protection guaranteed by the GDPR (Art. 44 GDPR). This reservation in favor of the other provisions of the GDPR makes it clear that Chapter V does not itself contain any sufficient legal basis for the transfer of personal data to recipients in a third country.^8^ The second-stage checklist can thus be found in Art. 45 et seq. GDPR.^9^

The following categories within this permissibility schema will be examined regarding their relevance to genomic data sharing in the context of German law: consent, privacy, security, adequacy, oversight, and future directions.

## Consent

Art. 9 GDPR defines the legal basis for the processing of sensitive personal data, including genomic data.^10^ The current general prohibition arose from the fact that the processing of sensitive personal data specifically affects fundamental rights and freedoms beyond Art. 8 of the Charter of Fundamental Rights (CFR), which binds EU institutions and member states when adopting or implementing the GDPR, Art. 51 (1) 1 CFR.^11^

Pursuant to Art. 9 (2) GDPR, the general prohibition of (1) does not apply in the cases of (a) to (j). Note three particularly relevant cases: Genetic data may be processed where explicit consent is provided (a). Genetic data may also be processed for scientific research purposes, provided that appropriate suitable and specific measures are taken (j). Finally, Art. 9 (4) authorizes member states to adopt further data protection provisions affecting the processing of genetic, biometric, and health data.

In Art. 27 (1) BDSG(neu), the national legislator has made use of the authorization under Art. 9 (2) (j) GDPR: By way of derogation from Art. 9 (1) GDPR, processing of special categories of personal data within the meaning of Art. 9 (1) GDPR shall also be permitted *without* consent for scientific research purposes where processing is necessary for these purposes and where the interests of the controller significantly outweigh the interests of the data subject.

In the previous Federal Data Protection Act, Art. 3 (9) defined special types of personal data (genetic and biometric data were, for example, not directly included) and Art. 4a (3)—in accordance with the DPD—defined explicit consent as the only possible legal basis for permission for processing. Thus, Art. 27 (1) BDSG(neu) represents a relaxation in favor of scientific research. However, where special legal regulations on data processing from sector-specific law apply, they take precedence over Art. 27 (1) BDSG(neu).^12^

In principle, according to the necessity test, Art. 27 (1) BDSG(neu) must provide for a balancing of interests. The controller must weigh their responsibilities to the project with the legitimate interests of the data subject. Accordingly, the processing of personal data is not necessary for scientific research purposes if said purposes could also be achieved with anonymized data. To avoid an “escape into privileged status”, only scientific research purposes are covered; an extension to other purposes cannot be foreseen.^13^ However, further processing of personal data for scientific research purposes is—according to Art. 5 (1) (b) GDPR—compatible with the original purposes of the first processing, so the legal basis in such cases may be based on the one already in use.^14^

In addition to the requirement of necessity, the interests of the controller in the processing must also considerably outweigh the interests of the data subject in the exclusion of the processing. Here, the legislator grants the interests of the data subject relative priority over the research interests of the controller, since only in the case of a significant predominance of research interests is the balance of interests in favor of the controller. Otherwise, the general constitutional principles for the balancing of interests apply.^15^ To transfer personal data to the third countries outside of the EU, the second step requires that the specific conditions for transfers to the third countries be complied with. In the absence of an adequacy decision pursuant to Art. 45 (3) GDPR, or of appropriate safeguards pursuant to Art. 46 GDPR, one potential reason to permit a transfer is—just as for the transfer per se—the explicit consent of the data subject to the proposed transfer [Art. 49 (1) (a) GDPR]. However, consent justifies the waiver of adequate data protection in the recipient country only if it complies with the requirements of Art. 4 No. 11 and Art. 7 GDPR, and the data subject has been duly informed beforehand about the risks of the transfer of data to a country without an adequate level of protection or suitable guarantees.^16^ The GDPR does not require a specific (e.g., written) form of consent but it should be prior (“proposed data transfer”)^17^ and voluntary, and the person concerned should be able to revoke it at any time *ex nunc*—although not retroactively.

Comprehensive information must include, among other things, the personal data or data categories and processes being consented to, the specific purpose of the transfer, the recipient’s identity, and the data’s destination.^18^ In addition, subjects must be informed of possible risks arising from the transfer—an abstract presentation is sufficient here^19^, and no detailed description of the legal situation in the recipient country is necessary.^20^ As consent must be explicit, silence or a mere opt-out cannot in principle be interpreted as consent.^21^ This implies stricter regulation compared to the corresponding regulation in the DPD, according to which there was still room under certain circumstances for consent through conclusive behavior.^22^ Against the background of the wording of Art. 49 (1) (a) GDPR (“the proposed data transfer”), blanket authorizations of transfers of personal data by the data subject to the third countries must be considered invalid.^23^

## Privacy

In the EU, the understanding of data protection has evolved within the traditional understanding of privacy.^24^ The DPD was influenced by German and French data protection laws^25^, with the former-anchoring data protection in human dignity and the right to informational self-determination^26^ and the latter in personal integrity^27^. Although the EU has long been committed to data protection as a fundamental right (Art. 8 CFR), for a considerable period, the Court of Justice of the European Union (CJEU) was reluctant in acknowledging the DPD’s objective as being the protection of such a right and emphasized its role solely in relation to achieving market integration. A change in the CJEU’s assessment was facilitated by the 2009 Lisbon Treaty.^28^ Accordingly, the GDPR removes most references to privacy and refers primarily to the right to data protection. It is, therefore, applicable to a broader range of personal data processing activities and grants individuals more rights in relation to a broader range of data categories. This enhanced control has both a conceptional function aimed at lessening the informational and power asymmetries between data subjects and data controllers, and an institutional function exercised through the subjective rights granted to individuals in relation to data controllers.^29^

When examining the permissibility of genomic data transfer for scientific research purposes to the third countries, data privacy, i.e., the sum of the data protection rights of data subjects, is particularly relevant when relying on Art. 9 (2) (j) GDPR as a legal basis for such processing, as it requires that the essence of the right to data protection be respected. Accordingly, the GDPR’s understanding of said essence—in particular specific rules on data subjects’ rights regarding transfers to third countries including informational rights when data are collected from the data subject [Art. 13 (1) (f) GDPR] and when data have not been obtained from the data subject [Art. 14 (1) (f) GDPR]—must be drawn on in connection with Art. 27 BDSG(neu).

Via the GDPR, EU law provides some limitations on data subjects’ rights in favor of scientific research—limitations on the data subject’s information rights [Art. 14 (5) (b) GDPR], right to erasure of personal data [Art. 17 (3) (b) GDPR^30^], and right to object where processing is in the public interest [Art. 21 (6) GDPR]. Art. 89 (2), (3) GDPR also permit member states to introduce limitations on certain data subject rights regarding scientific data processing. These data subject rights are the right to obtain information (Art. 15 GDPR), the right to correct data (Art. 16 GDPR), the right to restrict processing (Art. 18 GDPR), and the right to object (Art. 21 GDPR; however, note Art. 21 (6)). This exhaustive list limits member state derogation from the outset.

Based on the opening clause of Art. 89 (2) GDPR, Art. 27 (2) BDSG(neu) provides for a restriction of these rights if these are likely to seriously impair or render impossible the realization of research purposes. “Impossible” here means that there is no practicable way to achieve the research purposes without limiting the rights of the person concerned. For example, it might be impossible to achieve research purposes without restricting the Art. 15 GDPR right to access if the competent ethics committee for the protection of the person concerned would, otherwise, prohibit implementation of the project.^31^ Serious impairment can be assumed if the research purposes could only be achieved to a significantly lesser extent than would be possible were the rights of the persons concerned restricted. Said restriction must be necessary for the fulfillment of research purposes—it must be impossible to achieve them by any other means. It is sufficient that a specific right of the person concerned generally prevents the realization of one of the purposes listed in Art. 89 (2).^32^

Furthermore, according to Art. 27 (2) 2. sentence BDSG(neu), the data subject’s right to obtain information pursuant to Art. 15 GDPR does not exist if the data processing is necessary for the purposes of scientific research (so that it does not depend on a certain research purpose) and providing information would require disproportionate effort. In this context, the effort expended by the responsible body, e.g., the large number of data records concerned, must be weighed against the interest of the person concerned, i.e., the degree of endangerment of their right to data protection.^33^ Whether this derogation is actually based on any opening clause is a matter of heated debate.^34^

The role of data subject rights at the second stage of the examination of transfer permissibility will be touched upon in the section on adequacy below.

## Security measures

Security measures also play a decisive role in the two-stage permissibility test.

Several articles in the GDPR reference security measure based on Art. 89 (1), including Art. 9 (2) (j) GDPR itself as well as specific provisions of data subject rights, which must be drawn on when balancing involved interests via the application of said legal basis, such as Art. 14 (1) (f), Art. 14 (5) (b), and Art. 15 (2) GDPR.

Art. 89 GDPR does not constitute a separate authorization for the processing of personal data for scientific research purposes.^35^ In fact, it helps compensate for other areas of the GDPR which make such processing easier and also allows member states to adopt special rules for the processing of personal data for such purposes. This is done by laying down minimum requirements for processing for such purposes, regardless of other GDPR provisions which would, otherwise, facilitate such transfers.

The guarantees referred to in Art. 89 (1) GDPR must provide for technical and organizational measures, following the principle of data minimization in particular. For this reason, the only data that should be processed are those whose processing is necessary for the specific purpose of the processing. Here, particular attention must be paid to the amount of personal data collected, the scope of processing, the storage period, and accessibility.^36^ Art 89 (1) requires a graduated approach. Accordingly, before using personal data, the responsible body must check whether the intended purposes could also be achieved using anonymized data (compare recital 26 GDPR). If anonymization is ruled out (e.g., because subjects must be contacted again for health research purposes) or impossible, then pseudonymization must be considered.^37^ If neither anonymization nor pseudonymization is possible, the next question is whether data subject protection could be ensured by other proportionate measures, e.g., encryption or a non-disclosure agreement for the parties involved.

In addition, Art. 28 (3) (a), Art. 30 (1) (d) and (e), and Art. 30 (2) (c) define immediate security-related obligations regarding data transfers to the third countries, which, following the security measures arising from the legal basis, must also be fulfilled. Here, basic principles, particularly those related to data security, must be drawn on throughout data processing [Art. 5 (1) (f) GDPR].

According to Art. 27 (3) BDSG(neu), personal data processed for scientific research purposes must be anonymized. This represents a concretization of the principle of data minimization but it is, at this point, subject to a research-related reservation: The obligation to anonymize only arises once it is actually possible, taking into account the research purpose.^38^ This does not necessarily mean the obligation only arises once the entire research project is completed—it can already apply once the personal data are no longer required in an identified or identifiable form for the further course of research.^39^

Where longer term identification of the persons behind the data is necessary, pseudonymization should be preferred. It must, however, still be impossible to draw any direct conclusions about specific persons.^40^ Separation of personal data and identifiers can be implemented technically, e.g., by encoding data or using a linked file system—in which case that the key must be sufficiently secured and kept separately from the other files.^41^ Furthermore, no data can remain in the other system during separation, e.g., in the form of backup files.^42^

Art. 27 (3) BDSG(neu) refers to Art. 22 (2) 2. sentence BDSG(neu), which takes into account the fact that a research clause must provide for appropriate and specific measures to safeguard the fundamental rights and interests of the person concerned pursuant to Art. 9 (2) (j) GDPR. This may include further technical–organizational measures, such as subsequent verification of the processing of personal data or access restrictions, as well as measures aimed at raising awareness among those involved in data processing, or appointing a data protection officer in accordance with Art. 22 (2) BDSG(neu).

According to Art. 45 (2) (a) GDPR, security measures are also a basis for Commission adequacy decisions when examining data protection standards in the third countries. If no adequacy decision exists, various safeguards including security measures must be applied to enable transfers (compare the section on adequacy below).

## Adequacy

If its first-stage permissibility can be determined and it fulfills all relevant requirements outside Chapter V of the GDPR, a data transfer is still only permissible if it is also consistent with at least one of the grounds for authorization listed in Arts. 45 to 49 GDPR. A distinction must then be made between transfers to the third countries which guarantee an adequate level of data protection (Art. 45 GDPR) and those which do not (Art. 46, 47 or 49 GDPR).

The Commission is responsible for determining adequacy [Art. 45 (2), (3) GDPR]. For the Commission’s decision to be substantively lawful, an adequate level of protection must be maintained for data transfers to third countries.^43^ An assessment of the requirements for an adequate level of protection can be derived from recitals 102–104 and Art. 45 (2) GDPR. In addition, the CJEU judgment in *Schrems*—particularly recitals 72 and 73—must be observed, this ruling being the basis for the substantial extension of the conditions for adequacy.

An adequate level of data protection is, therefore, determined in substantive and legal terms primarily via an assessment of the rule of law and respect for human rights and fundamental freedoms in the third country, any applicable data protection regulations, and the case law of the CJEU. The adequate level of data protection is, therefore, a level essentially equivalent to that guaranteed within the EU. For the substantive requirements to be effective and enforceable, there must also be independent supervision of data protection via administrative and judicial remedies by the parties concerned and by supervisory authorities. It also follows from *Schrems* that, to determine the adequate level of protection, particular attention must be paid to the guarantees of the CFR, since regulations serve to implement Art. 8 CFR.

It can, therefore, be stated that an adequate level of protection is one which respects the fundamental values of the EU and the protection of human and fundamental rights; this applies in the context of data protection law specifically to Art. 8 CFR, supported by the wording that the continuation of the level of protection must be guaranteed and those concerned must be granted effective and enforceable rights.^44^ The concretization of Art. 8 CFR via the GDPR through the operational declaration of specific rights is, therefore, decisive for determining an adequate level of protection. A legitimate Commission decision allowing the transfer of personal data to a third country must, therefore, be measured against these requirements (recital 102 GDPR).

If there is no Commission adequacy decision, other legal bases could be drawn on to allow data transfer. It should, however, be noted that, for example, standard contractual clauses and codes of conduct each also require a Commission decision should they be approved and applied throughout the EU. Given the criticism that actual adequacy decisions are often not based on factual adequacy in the third country^45^, other legitimate grounds for processing might also not prevent the level of protection guaranteed by the GDPR from being undermined (Art. 44 GDPR). Ultimately, as long as adequacy decisions exist, incentives to negotiate stronger data protection rules for transfers including private law instruments will remain limited.^46^

The prevailing opinion denies the applicability of the compelling legitimate interest of the controller as defined by Art. 49 (1) 2. sentence as a legitimate ground for processing genomic data, since processing for such purposes regularly fails to fulfill the conditions that the transfer not to be repetitive and only concern a limited number of data subjects.^47^

## Oversight

Transfer of genomic data for scientific research purposes to third countries, comparable to other member state laws, involves several oversight bodies, institutional review boards, research ethics committees, and data protection authorities.^48^ EU law requires that member states allow for the processing of genetic and health data for research purposes only when suitable or appropriate safeguards are in place. Such a safeguard could be the involvement of a research ethics committee, as some member states have demonstrated.^49^

As adjustment in sector-specific laws is still on the cards, the focus here should be on the role of data protection supervisory authorities in the transfer of data to third countries, in the context of the interaction between the GDPR and the BDSG(neu).

According to recital 122 GDPR, each supervisory authority should be competent in the territory of its own member state to exercise the powers and perform the tasks conferred on it in accordance with the Regulation. This should, however, also include processing which affects data subjects in the territory of the supervising authority or processing carried out by a controller or processor not established in the EU targeting data subjects residing in its territory. In such cases, supervisory authorities’ powers should include handling complaints lodged by data subjects, conducting investigations into the application of the GDPR, and promoting public awareness of the risks, rules, safeguards, and rights involved in personal data processing.

Recital 116 GDPR notes that supervisory authorities may find themselves unable to pursue complaints or conduct investigations relating to activities outside their borders, especially when personal data move outside the EU. Cross-border cooperative efforts may also be hampered by insufficient preventative or remedial powers, inconsistent legal regimes, and practical obstacles like resource constraints. Closer cooperation between these authorities must be promoted, to help them exchange information and carry out investigations with their international counterparts. To develop international cooperation mechanisms for the enforcement of legislation for the protection of personal data, the Commission and supervisory authorities should exchange information and cooperate in activities related to the exercise of their powers with competent authorities in third countries, based on reciprocity and in accordance with the Regulation.

Data subjects may lodge a complaint with a supervisory authority if they think the processing of their personal data are contrary to the GDPR (Art. 77 et seq. GDPR). The independent data protection authorities of member states must then review the lawfulness of the transfer and suspend it if they find the GDPR is violated (Arts. 45, 58 GDPR, Art. 21 (1) BDSG(neu)). In *Schrems*, the ECJ derives this right directly from Article 8 (1), (3) CFR.

In any case, although legal protection prospects exist for individuals, promising administrative and legal remedies against data breaches often become drawn-out and ineffective. Due to the second-stage permissibility of data transfers to third countries based on Chapter V of the GDPR, and because adequacy decisions of the EU Commission often provide prominent legal bases for transfers approved EU wide, cases must pass several appeal stages before a final decision can be made (Art. 77 et seq. GDPR). A special feature of the proceedings in Germany is that, in the event that the supervisory authority suspends the proceedings due to the assumption of a violation of the GDPR, it itself as a party submits the case to the Federal Administrative Court for decision [Art. 21 (3), (4) BDSG(neu)]. The technical expertise of the supervisory authority is assumed to be conducive to the court proceedings [Art. 53 (2) GDPR]. Although, in national legal proceedings, the Federal Administrative Court is thus responsible at the first and last instances [Art. 21 (3) BDSG(neu)], proceedings can still be lengthy and there remain plenty of hurdles to overcome, from national data protection supervision to the CJEU; altogether, legal protection against the transfer of personal data at a potentially inadequate level of data protection amounts to a preliminary ruling procedure before the CJEU pursuant to Art. 267 TFEU with all its limitations (e.g., damages claims and the unclear powers of supervisory authorities to suspend single transfers when dealing with complaints in light of the primacy of EU law^50^).

## Future directions

The GDPR recognizes, as a basic legitimate interest, the processing of personal data for scientific research purposes, the importance of which is stressed in EU law via, for example, the promotion of scientific research and progress (Art. 3 (3) 1 TEU) and the creation of a European Research Area (Art. 179 (1) TFEU). Research purposes should also be understood in light of the CFR, primarily Art. 11 CFR (freedom of expression and information) and Art. 13 CFR (freedom of art and science). However, these fundamental rights are not granted without restriction, but are, in turn, subject to restrictions under national and EU law, in particular the right to protection of personal data pursuant to Art. 8 CFR. Against this background, Art. 89 GDPR balances the conflicting interests of data controllers and the general public on one hand and the data subjects on the other.

From a national, constitutional point of view, research work using personal data is caught between the freedom of science and research guaranteed in Article 5 (3) of the Basic Law and the right of individuals to informational self-determination under Article 2 (1) in conjunction with Article 1 (1) of the Basic Law.^51^

In principle, fundamental Union rights apply within the scope of EU law. Since secondary legislation now largely regulates data protection in member states, there are a few areas in which the EU fundamental right to data protection does not apply. At the same time, the opening clauses provide for an appropriate balance between the various fundamental rights protected by the Union’s legal order. Within these margins, member states must respect their fundamental national rights in addition to fundamental Union rights.

As the GDPR is considered to be partially incomplete and somewhat simplified, with, for example, responsibilities regarding health-relevant data transfers to third countries often remaining unclear, only a partial primacy of the application of EU law can be contested. How far the application priority actually extends must be determined on a case-by-case basis, thus influencing the GDPR’s harmonization effect.^52^

Regarding general implementation, the BDSG(neu) has been subject to criticism. Critics have pointed out that, primarily due to restricting the rights of persons concerned^53^, it leads to a weakening of the previous data protection level and—contrary to the objective of the GDPR—creates legal uncertainty. Its particular use of opening clauses is also viewed critically, because the comprehensive continuation of the previous federal legal provisions aimed at maintaining high levels of protection endangers the harmonization aims of the GDPR and thus makes the law more difficult to apply.^54^ In contrast, harmonization of the second-stage permissibility of transfers to third countries has been partially successful, but uncertainties regarding interpretation might outweigh the benefits for many years.

The main reasons states conclude data transfer agreements are often economic—as the GDPR already points out (see its title): not only to protect personal data, but also to enable their free movement, Art. 1 (1) GDPR. It is unfortunate that the Commission’s adequacy decisions occasionally fail to strike a proper balance between data protection and free movement of data in a world where data increasingly act as currency, allowing individuals to participate in various inherently cross-border and global activities especially in the areas of medical research and public health. Efforts are still needed to promote international research so as to prevent patients from having to pay the ultimate price, which, to quote the GDPR, would inherently be “inadequate”.

## Footnotes

1 Pauly in: Paal/Pauly, Datenschutz-Grundverordnung, Bundesdatenschutzgesetz, 2nd ed. 2018, DSGVO Art. 44 para. 3.

2 N.B.: the GDPR is not applicable to the processing of data of deceased persons (recital 27 GDPR).

3 Arzneimittelgesetz in der Fassung der Bekanntmachung vom 12. Dezember 2005 (BGBl. I p. 3394), das zuletzt.durch Artikel 1 des Gesetzes v. 18. Juli 2017 (BGBl. I p. 2757) geändert worden ist.

4 Regulation (EU) No 536/2014 of the European Parliament and of the Council of 16 April 2014 on clinical trials on medicinal products for human use, and repealing Directive 2001/20/EC.

5 Pauly in: Paal/Pauly, 2nd ed. 2018, BDSG Sect. 27 para.4.

6 Compare prevailing opinions, e.g., Ambrock/Karg, Ausnahmetatbestände der DS-GVO als Rettungsanker des internationalen Datenverkehrs? ZD 2017, 154, 155; Schröder in: Kühling/Buchner, 2nd ed. 2018 DS-GVO Art. 44, para. 20. Compare, on the two-stage permissibility test, a short German version: Molnár-Gábor, Die Regelung der wissenschaftlichen Forschung in der DS-GVO, in: J Taeger/Deutsche Stiftung für Rechtsinformatik (eds.), Rechtsfragen digitaler Transformationen—Gestaltung digitaler Veränderungsprozesse durch Recht, Tagungsband Herbstakademie 2018.

7 In the case of individual researchers, the important question would be whether they receive data by virtue of their position in an international organization. If they do not, transfers to such researchers could still qualify as transfers to a third country. On the interpretation of “to”, see: Moos/Zeiter, in: Moos/Schefzig/Arning, Die neue Datenschutz-Grundverordnung, 2018, Internationale Datenübermittlungen, p. 290–1, para. 7–9.

8 Kamp in: BeckOK DatenschutzR Wolff/Brink, 24th ed. 01.02.2017, DS-GVO Art. 44 para. 21.

9 Adequacy decision; EU standard data protection clauses; binding corporate rules; approved code of conduct; approved certification of the data importer; consent of all data subjects; transfer for the performance of a contract; transfer for public interest; transfer for assertion of legal claims; transfer to protect vital interests; transfer from a public register; transfer for safeguarding legitimate interests, Wybitul/Ströbel, Übermittlung personenbezogener Daten in Drittländer, ZD 2017, 503. Due regard must also be given to Art. 49 (5) GDPR, an opening clause giving member states the option of limiting the transfer of special categories of personal data to the third countries.

10 The use of the legal basis in Art. 6 GDPR must be excluded, as this would circumvent the high barriers of Art. 9 GDPR, Schantz, in: Schantz/Wolff, Das neue Datenschutzrecht, C.H. Beck 2017, para. 705.

11 Supplemented in: Kühling/Buchner, DS-GVO Art. 9, para. 16.

12 Such special legal regulations may be enshrined in future law, such as medical law (e.g., Medicines Act, see above, Act on Genetic Diagnostics: Gendiagnostikgesetz v. 31. Juli 2009 (BGBl. I p. 2529, 3672), das zuletzt durch Artikel 2 Absatz 1 des Gesetzes vom 4. November 2016 (BGBl. I p. 2460) geändert worden ist).

13 Schantz, in: Schantz/Wolff, para. 1346.

14 Deutscher Bundestag, 18. Wahlperiode, Gesetzentwurf der Bundesregierung, Entwurf eines Gesetzes zur Anpassung des Datenschutzrechts an die Verordnung (EU) 2016/679 und zur Umsetzung der Richtlinie (EU) 2016/680 Datenschutz-Anpassungs- und -Umsetzungsgesetz EU—DSAnpUG-E, BT-Drs. 18/11,325, 24.02.2017, p. 99.

15 Pauly in: Paal/Pauly, BDSG Sect. 27 para. 7–8.

16 Working Party on the Protection of Individuals with Regard to the Processing of Personal Data, Transfer of Personal Data to Third Countries: Applying Articles 25 and 26 of the EU Data Protection Directive (WP 12, July 1998), p. 26.

17 Article 29 Working Party, Working document on a common interpretation of Article 26(1) of Directive 95/46/EC of 24 October 1995 (WP 114, 25.11.2005), p. 12.

18 Pauly in: Paal/Pauly, DS-GVO Art. 49, para 7–10.

19 Schröder in: Kühling/Buchner DS-GVO Art. 49, para. 15.

20 Lange/Filip in: BeckOK DatenschutzR DS-GVO Art. 49, para. 8.

21 WP 114, p. 12.

22 WP 12, p. 26.

23 WP 114, p. 14.

24 Molnár-Gábor, Data Protection, in: Max Planck Encyclopedia of Comparative Constitutional Law (Oxford University Press 2017).

25 Heil, Directive 95/46/EC of the European Parliament and the Council, in: Büllesbach, Poullet, and Prins (eds), Concise European IT Law (Kluwer Law International 2010), p. 9.

26 Population Census Decision 1 BvR 209/83 (15 December 1983) BVerfG 65, 1 (Ger).

27 Loi Portant Création d’une Couverture Maladie Universelle (23 July 1999) Décision No. 99–416 DC (Fr).

28 Art. 16 TFEU, Art. 6 (1) TEU.

29 Linskey, The Foundations of EU Data Protection Law (Oxford University Press 2015), p. 11 et seq.

30 Compare the controversial discussion of the German implementation of the right to erasure, Paal in: Paal/Pauly, BDSG Sect. 35, para. 2 et seq.

31 BT-Drs. 18/11,325, p. 99.

32 Buchner/Tinnefeld in: Kühling/Buchner DS-GVO Art. 89, para. 24.

33 Däubler in: DKWW BDSG aF Sect. 33 para. 29; BT-Drs. 18/11,325, p. 99.

34 Critically assessed by Schantz in: Schantz/Wolff, para. 1357. The opening clause Art. 23 (1) i GDPR might be drawn on, which allows member states to impose restrictions on certain rights and obligations to which data controllers or data processors are subject under national law, if this constitutes a measure which safeguards the rights and freedoms of other persons.

35 Pauly in: Paal/Pauly, DS-GVO Art. 89, para. 1.

36 Id, para. 11–12.

37 Schaar, Geänderte Vorgaben für die Wissenschaft, ZD 2016, 224, 225.

38 Plath/Frey in: Plath BDSG aF Sect. 40, para. 9.

39 Paal, in : Paal/Pauly, BDSG Sect. 27 para. 16 et seq.

40 Gola/Schomerus BDSG aF Sect. 40, para. 14.

41 Id.

42 Gerling in: Roßnagel DatenschutzR Chap. 7.10, para. 11.

43 N.B.: the decision on adequacy does not relate to the concluding processing of data in the third country but instead solely to the data transfer.

44 CJEU, Judgment in Case *Schrems v Data Protection Commissioner*, case C-362/14 [2015], EU:C:2015:650.

45 Molnár-Gábor/Kaffenberger, EU-US Privacy Shield—Ein Schutzschild mit Löchern? Bedeutung für den Austausch von personenbezogenen Daten in der medizinischen Forschung, ZD 2016, 18.

46 Molnár-Gábor/Kaffenberger, Privacy Shield—Bedeutung des Angemessenheitsbeschlusses der EU-Kommission. Rechtsschutz bei der transatlantischen Verarbeitung personenbezogener Daten ZD 2018, 162, 167.

47 Compare only: Pauly in : Paal/Pauly, DS-GVO Art. 49 para. 28 et seq.

48 See Reichel, Oversight of EU medical data transfers—an administrative law perspective on cross-border biomedical research administration, Health Technol. (Berl.) 2017; 7(4):389–400.

49 Compare Swedish law: Id., Fn. 20: Sect. 19 Personuppgiftslag (1998:204) [Personal Data Act, implementing the EU data Protection Directive], which refers to lag (2003:460) om etikprövning av. forskning som avser människor [Act concerning the Ethical Review of Research Involving Humans].

50 For measures on interim legal protection, compare Art. 21 (6) 1 BDSG(neu) in connection with Art. 47 (6) of the Law on Administrative Court Proceedings (VwGO). VwGO: Verwaltungsgerichtsordnung in der Fassung der Bekanntmachung vom 19. März 1991 (BGBl. I p. 686), die zuletzt durch Artikel 5 Absatz 2 des Gesetzes vom 8. Oktober 2017 (BGBl. I p. 3546) geändert worden ist.

51 Grundgesetz für die Bundesrepublik Deutschland in der im Bundesgesetzblatt Teil III, Gliederungsnummer 100-1, veröffentlichten bereinigten Fassung, das zuletzt durch Artikel 1 des Gesetzes vom 13. Juli 2017 (BGBl. I p. 2347) geändert worden ist.

52 Roßnagel (ed.) Europäische Datenschutz-Grundverordnung (Nomos 2017), p. 78, Sect. 2, para. 33–34.

53 Jensen, Kritik von Sachverständigen zum derzeitigen Entwurf des DSAnpUG-EU, ZD-Aktuell 2017, 05596.

54 Kühling, Neues Bundesdatenschutzgesetz—Anpassungsbedarf bei Unternehmen, NJW 2017, 1985, 1986. Roßnagel, Nationale Anpassung des Datenschutzrechts, DuD 2018, 471–472.

